# Community based interventions for the primary prevention of cardiovascular disease in women living in rural, regional and remote areas: a scoping review

**DOI:** 10.1186/s12913-026-14192-z

**Published:** 2026-02-17

**Authors:** Madison Frith, Mary Rose Angeles, Feby Savira, Sean Randall, Crystal Man Ying Lee, Rachel Huxley, Suzanne Robinson

**Affiliations:** 1 Institute for Health Transformation, Deakin University, Geelong, Australia; 2 Faculty of Health, Deakin University, Geelong, Australia; 3 Curtin University, Perth, Australia

**Keywords:** Cardiovascular disease, Primary prevention, Primary care, Community based interventions, Women's health

## Abstract

**Background:**

Cardiovascular disease (CVD) is the leading cause of death among women worldwide, yet prevention has historically centered on male risk profiles. For women living in rural, regional and remote settings, compared with metro settings, cardiovascular risk is higher because of the intersectionality between gender and geographical determinants such as limited access to timely care. Community-based and primary-care interventions show promise in addressing modifiable risk factors, but consolidated, gender-specific evidence for non-metropolitan contexts remains limited.

**Objective:**

This scoping review synthesises community-based interventions for primary CVD prevention among rural and regional women, mapping current approaches and gaps to inform future practice and policy toward equitable cardiovascular outcomes.

**Methods:**

A scoping review was conducted on both quantitative and qualitative publications in Medline Complete, Embase, CINAHL Complete, APA PsychINFO and AMED databases.

**Conclusion:**

This review of community-based interventions for primary CVD prevention in rural, regional and remote women found multicomponent programs (education plus coaching, goal-setting and self-monitoring) delivered in-person and in groups were more effective than single-component approaches. However, most interventions were not gender-specific, rarely co-designed, and seldom included gender-based analyses, reflecting persistent gender bias. Future research should embed co-design initiatives with women living in rural, regional and remote communities to address cardiometabolic risks and rural access barriers. Gender-disaggregated outcomes and implementation measures should also be routinely reported.

**Supplementary Information:**

The online version contains supplementary material available at 10.1186/s12913-026-14192-z.

## Introduction

Cardiovascular disease (CVD) remains the leading cause of morbidity and mortality worldwide, accounting for approximately 41.7% of all deaths globally in 2023 [1]. Broadly encompassing disorders such as coronary heart disease, stroke, cerebrovascular disease, and peripheral arterial disease, CVD imposes a significant burden on individuals, health systems, and economies [2, 3].

Although historically perceived as a predominantly male disease, CVD is the leading cause of death among women globally [4]. Women face unique life-risk factors, including those related to pregnancy (e.g., preeclampsia, gestational diabetes), menopause, and hormonal influences [2, 4]. Despite experiencing lower rates of CVD during their reproductive years, women have a higher mortality and poorer health outcomes following acute cardiovascular events compared to men [2, 4]. In Australia, more than half a million women are living with CVD placing them at substantially increased risk of premature mortality [5]. Clinical management often reflects sex and gender disparities, with women being less likely to undergo diagnostic procedures, receive evidence-based treatments [6], or be referred to cardiac rehabilitation [2, 7]. Furthermore, structural, systemic, and implicit biases within healthcare systems exacerbate these inequities [7–9]. This reflects intersectionality, which is a theoretical framework that examines how multiple, interconnected systems of power work together to shape peoples lived experiences and outcomes, particularly for socially marginalised groups [10]. In the context of women’s CVD prevention, an intersectional lens highlights how overlapping social and structural forces can compound disadvantage and increase CVD risk. Applying this lens, gender also intersects with geography: women living in rural, regional, and remote areas experience significantly higher CVD-related morbidity and mortality than women in metropolitan areas [5]. Factors contributing to this inequity include limited access to healthcare services, workforce shortages, geographic isolation, and socioeconomic disadvantage [5]. Moreover, rural healthcare systems often face systemic barriers to implementing evidence-based interventions, such as infrastructure limitations and fragmented care networks [5]. This can lead to delayed recognition of acute cardiovascular symptoms, barriers to early diagnosis and treatment, and reduced access to cardiac rehabilitation services [6].

Research has also demonstrated that women living in rural areas may experience higher rates of undiagnosed disease, greater delays in treatment, sub-optimal treatment pathways, and poorer follow-up care outcomes [6]. Understanding and addressing the intersectionality between gender and geography is critical to improving cardiovascular outcomes in women living in rural, regional, and remote areas.

In this review, the term gender (rather than sex) is used when referring to women and men to be inclusive of people identifying as women and gender-diverse people. It is also used to reflect that CVD risk and prevention are shaped by social roles, relations, and power structures, not biology alone. Gender is defined as a social construct, the socially constructed roles, behaviours, expressions, and identities associated with being a woman, man, or gender-diverse person, which varies across contexts and can change over time [11].

Major modifiable risk factors for CVD include smoking, obesity, diabetes, hypertension, and hyperlipidemia [4]. Each of these factors contribute significantly to the development of atherosclerosis and other cardiovascular complications through mechanisms such as endothelial dysfunction, inflammation, and thrombosis [2]. Importantly, these risk factors are also influenced by behavioural, commercial, and socioeconomic determinants, underlining the importance of public health and primary health care approaches to CVD prevention.

Community-based and primary care interventions have emerged as promising strategies to address these risk factors and bridge the gap between health outcomes for women compared to men. Evidence from both high-income and low- to middle-income countries suggests that community-led programs can effectively increase population knowledge, promote healthier lifestyle behaviours, and reduce CVD risk factors​ [12]. For example, in rural settings, integrating community health workers, telehealth solutions, and culturally tailored programs has shown promise in overcoming access barriers and improving outcomes [12, 13]. Evidence also suggests that gender-specific interventions are more effective than non-tailored evidence [14] with women shown to have different preferences to receiving an intervention compared to men [15].

Whilst there are many interventions to address CVD risk, there is limited consolidated evidence on gender-specific interventions in rural, regional, and remote areas in the context of CVD prevention. By synthesising existing literature of community-based interventions targeted at primacy CVD prevention in women living in these areas, this review seeks to highlight current interventions, identify gaps, and inform future efforts toward achieving equitable cardiovascular health outcomes. Therefore, the aim of this scoping review is to examine community-based interventions targeting CVD prevention via addressing modifiable risk factors for women residing in rural and regional areas. Specific research questions are as follows:

1. Which CVD risk factors and outcomes are most examined in community-based interventions?
2. How does rurality impact intervention outcomes?
3. How does gender impact intervention outcomes?
4. How is co-design used in designing interventions?

## Methods

### Search strategy and study selection

A scoping review was conducted on quantitative and qualitative publications on interventions targeting the primary prevention of community-based CVD (Box 1). We searched publications (from inception to 26 June 2025) in Medline Complete, Embase, CINAHL Complete, APA PsychINFO and AMED databases, supplemented by internet searches and checking of reference lists for grey literature and other relevant publications (see Additional File 1: Supplementary Table 1 for search strategy). Review protocol is available upon request to the corresponding author.

**Box 1:** Inclusion and exclusion criteria for scoping review of published studies on community based primary cardiovascular disease (CVD) preventive interventions based in rural, regional, and remote areas

Criteria	Inclusion criteria	Exclusion criteria
Participants	• Aged ≥ 18 years. • Participants living in rural, regional, and remote areas.	• Aged < 18 years. • Participants only residing in metropolitan or urban areas.
Intervention	• Primary and community care interventions for the primary prevention of CVD for females, or males and females. Interventions that are at least in part delivered in rural, regional, or remote areas. • Interventions are in-person or digital but interact with a wider community or a hybrid of in-person and digital.	• Secondary preventive interventions for CVD in females. • Interventions that only target males. • Interventions that are only delivered in metropolitan or urban areas. • Interventions that are digital only and has no community interaction.
Comparator	• Any.	• Not applicable.
Setting	• Primary care.	• Secondary or tertiary care.
Study type	• Randomised or non-randomised trials, observational studies, interrupted time-series or repeated measures studies, embedded/implementation trials, and government evaluation reports. • Qualitative and quantitative studies.	• Opinion pieces and conference abstracts. • Study protocols. • Systematic and other reviews.
Outcomes	• CVD or related risk factor outcomes. • Participant experiences. • Reach, adherence/attendance of an intervention. • Cost effectiveness of intervention.	• No CVD or related risk factor outcomes.
Language	• English.	• Language other than English.

To ensure a comprehensive search, the review included synonyms of CVD using Embase Emtree as well as MeSH terms for CVD in the search strategy. The search also focused on studies that addressed the primary prevention of CVD, that is the prevention of CVD occurring in the first instance through managing risk factors [16]. “Community-based” was used as an explicit search concept, defined broadly to include interventions that view the community as a setting, target population, agent of change, or resource. These interventions are diverse and typically focus on enhancing the health and well-being of local groups through approaches such as health promotion activities, community mobilisation, health education, behaviour change programs, environmental modifications, and policy or advocacy initiatives directed at individuals or entire communities [17]. All interventions that incorporated at least one or more approaches were included for screening. Purely digital interventions were excluded to maintain the review’s focus on interventions that incorporate a face-to-face component. For this paper, community-based interventions focused on the primary prevention of CVD, that is preventing CVD occurring in the first instance. Interventions were required to take place in a primary care or community setting and not a secondary or tertiary setting. This included community health workers, peer support workers, nurses, allied health professionals, general practitioners, and pharmacists. Hospital-based interventions were excluded.

### Screening

Screening was conducted using Research Screener, a cloud-based web platform that uses an algorithm to semi-automate abstract screening for reviews [18]. It assisted with study selection by organising citations and supporting title/abstract and full-text screening. The tool facilitated structured screening workflows, documentation of inclusion/exclusion decisions, and transparent tracking of screening progress across reviewers. Two authors (MF, MRA) conducted screening of the title and abstracts followed by full text. Reasons for exclusion included studies where the target population was not inclusive of both males and females, focused on children, evaluated digital-only interventions, or were study protocols. The authors resolved disagreements through collaborative discussions referring to the inclusion and exclusion criteria. The lead author (MF) had the final say upon disagreements.

### Data extraction and synthesis

The quality of the included studies was assessed using the JBI methodology for scoping reviews [19].Two authors (MF, MRA) conducted data extraction from the studies, with one reviewer (MF) verifying all extracted information. We collected general study details (such as author(s), title, publication year, country, study design, sample size, characteristics, setting); CVD sub-types targeted; CVD risk factors targeted; mode of intervention delivery (in-person, virtual, or hybrid); type of intervention (e.g. health education, lifestyle counselling); co-design of interventions (if utilised); outcomes measured and reported gender differences. All findings were narratively synthesised. See Supplementary Table 2 for Scoping Review (PRISMA-ScR) Checklist.

## Results

We identified 1,034 potentially relevant publications through database searches. After removing duplicates, 762 records remained for screening. Of these, 656 were excluded as not relevant to our research question. We then assessed the full text of 106 articles against our inclusion criteria, resulting in 62 articles being included in the final review (see Additional File 1: Supplementary Fig. 1 for PRISMA flow diagram), including 53 quantitative, 5 qualitative and 4 mixed methods studies. Table 1 provides a summary of results (insert Table 1 at the end of the document here).

**Table 1:** Summary of included studies

Study	Country	Study type	*N* (% female)	Mean (SD) age (years)	Setting	Prevention target	Risk factor	Mode	Intervention type
Ammerman 2003 [20]	USA	RCT	468 (71%)	55	Rural	CVD risk factor	Hypertension	Hybrid (in person, online, telephone)	Food education, counselling
Arikan 2011 [21]	Turkey	Semi-experimental	Phase 1: 2766 (59.6%) Phase 2: 778 (55.1%)	43.9 (12.3)	Semi-rural	CVD risk factors	CVD risk factors	Hybrid (in person, telephone)	Risk factor awareness and education on heart healthy behaviours
Bekwelem 2012 [22]	USA	Cross sectional	126 (60%)	47.7 (14.8)	Rural	CVD risk factors	CVD risk factors	In person	Coaching/counselling, health survey
Cappuccio 2006 [23]	Ghana	Cluster randomaised	1013 (61.2%)	55	Rural and semi-urban	CVD risk factor	Hypertension and salt intake	In person	Health education
Carrington 2015 [24]	Australia	Observational	530 (62%)	54 (14)	Regional	CVD risk factors	CVD risk factors and diabetes risk factors	Hybrid (telephone, email, in person)	Health education, behaviour change, screening
Cornell 2009 [25]	USA	Observational	N/R (100%)	N/R	Rural	CVD risk factors	CVD risk factors	In person	Environmental changes, physical activity training, nutrition education and cooking classes, smoking cessation
Fahs 2013 [26]	USA	RCT	274 (100%)	51 (7.6)	Rural	CVD risk factors	Diet, physical activity and smoking	Hybrid (in person, online)	Health coaching
Folta 2009 [27]	USA	RCT	91 (100%)	I: 57.8 (9.8), C: 57 (8.1)	Rural	CVD risk	Obesity	Hybrid (in person, video)	Physical activity, food education
Folta 2019 [28]	USA	RCT	194 (100%)	58.6 (9.5)	Rural	CVD risk	Obesity, poor diet, physical inactivity	In person group classes	Education, physical activity
Frank 2008 [29]	USA	Observational	120 (70%)	45.8	Rural	CVD risk and secondary prevention for CVD, stroke and diabetes	Obesity, hypertensionpressure, diabetes, poor diet, physical inactivity, smoking/alcohol use	In-person, group and individual within church setting	Health education, screening, and counseling
Greer 2011 [30]	USA	RCT	60 (100%)	58.0 (12.4)	Rural	CVD risk and secondary prevention	Hypertension, high sodium intake, overweight/obesity, poor treatment adherence	In-person, group-based	Health education and counselling (culturally tailored)
Hageman 2014 [31]	USA	Community based clinical trial	289 (100%)	56.4 (6.3)	Rural	CVD risk	Hypertension	Hybrid (in person, telephone, video, print)	Health counselling, physical activity, health education
Hageman 2010 [32]	USA	Community based clinical trial	225 (100%)	57.9 (5.6)	Rural	CVD risk factors	Hypertension, low fitness, high cholesterol	Hybrid (print, online)	Health education via newsletters, screening
Holben 2017 [33]	USA	Observational	74 (77.1%)	50.1 (8.8)	Rural	CVD risk	Obesity/adiposity, dyslipidemia, elevated glucose, hypertension, low fitness; behavioral risk (sedentary lifestyle, stress)	In-person, group + individual sessions	Counseling/education + supervised exercise + stress management (non-technology lifestyle program)
Hoy 2005 [34]	Australia	Observational	1070 (57%)	37.4 (15)	Remote	CVD risk and renal prevention	Elevated BP, albuminuria/proteinuria, diabetes, overweight/obesity patterns, smoking and alcohol use	In person	Screening + education + treatment/medication management
Husain 2025 [35]	Germany	Non-randomised controlled longitudinal	175 (68%)	I: 58.3 (8.0),C: 53.3 (9.6)	Rural	Primary prevention of NCDs including CVD	Poor diet, physical inactivity, overweight/obesity, stress, social isolation	In-person (group seminars + individual coaching)	Lifestyle education and behaviour change
Iso 1998 [36]	Japan	Non-randomised comparative longitudinal	Full intervention: 3217 (NR) Minimal intervention: 1,468 (NR)	N/R	Rural	Stroke	Hypertension (including salt intake reduction, excessive alcohol)	In-person screenings, clinic referrals, home visits, community classes, local media broadcasts	Public health screening, health education, and counselling
Keyserling 1999 [37]	USA	RCT	467 (71%)	55	Rural	CVD risk	Obesity, high fat intake, low physical activity, dyslipidemia	Hybrid (in person group sessions, telephone)	Behavioral lifestyle intervention, counselling
Kotwani 2014 [38]	USA	RCT	225 (47.2%)	51.2	Rural	CVD risk	Hypertension	In person	Health counselling, education
Kramer 2010 [39]	USA	Non-randomised prospective pretest-posttest	81 (87.65%)	52.9	Urban, suburban, rural	CVD risk	Diabetes, Overweight/obesity & physical inactivity (via weight-loss/PA goals); dyslipidaemia, hypertension, hyperglycaemia	In-person, small-group sessions	Behavioral lifestyle education/counselling
Kurnia 2022 [40]	Indonesia	Quasi-experimental, pre test posttest	41 (83%)	64.4 (6.7)	Rural	CVD risk	Hypertension and related lifestyle factors (medication adherence, diet incl. salt intake, physical activity)	In person (group lecture at public health center)	Health education
Landy 2011 [41]	USA	Observational	1694 (59%)	45–64 years	Rural	CVD risk	Hypertension; hyperglycaemia/diabetes; dyslipidaemia (LDL/HDL); overweight/obesity; smoking	In person (one-day rural outreach health fairs)	Screening + health education/counselling
Lilly 2014 [42]	USA	Pre post pilot intervention; quasi-experimental	81 (90.2%)	52.8 (12.2)	Rural/semirural	CVD risk	Poor diet, physical inactivity, excess weight (weight monitored), high stress	In person (group sessions)	Behavioral problem-solving training + lifestyle education/counseling
Lo 2020 [43]	USA	Secondary analysis	Intervention: 101 (82.1%) Control: 93 (83.4%)	I: 49.1 (16.6); C: 45.1 (14.0)	Rural	CVD risk	Overweight/obesity; physical inactivity; poor diet quality	In person (group classes/activities)	Behavioral lifestyle program (exercise + diet education; community capacity building)
Lo 2021 [44]	USA	Cluster RCT	194 (100%)	58.9 (9.5)	Rural	CVD risk	Physical inactivity; poor diet quality; overweight/obesity (BMI)	In person (group classes and activities)	Multilevel behavioral lifestyle program (exercise + diet education; social support; community engagement)
Low 2007 [45]	USA	Observatonal	NR (100%)	N/R	Rural	CVD risk	Diabetes; obesity; physical inactivity; tobacco use; hyperlipidaemia; hypertension	In person (community sessions; clinic-based visits)	Health education/outreach; chronic disease management (multidisciplinary)
Manuel 2016 [46]	Canada	Evaluation	32 (100%)	N/R	Rural and urban	CVD risk and stroke risk reduction	Hypertension, hypercholesterolemia, diabetes, obesity, smoking, sedentary lifestyle, diet quality	In person (group education + interactive activities)	Health education/counselling
Marigliano 2016 [47]	USA	Quasi-experimental	62 (100%)	55	Rural	CVD risk	Physical inactivity, overweight/obesity, hypertension, dyslipidemia, smoking	In-person assessments & talk + self-directed walking	Physical activity, brief education
Mitchell 2018 [48]	USA	One-group pretest–posttest community program	40 (95%)	N/R	Rural	CVD risk	Physical inactivity, diet, obesity, hypertension, diabetes	In person (group sessions)	Group exercise classes + health education/counselling
Nguyen 2011 [49]	Vietnam	Implementation with observational follow up	1180 (53.8%)	65.1 (14.1)	Rural	CVD risk	Hypertension, diet, alcohol consumption, smoking, physical inactivity, overweight/obesity	In person (monthly clinic check-ups; community IEC sessions; on-site training)	Pharmacotherapy, lifestyle counselling and education, health system training
Nguyen 2012 [50]	Vietnam	Quasi-experimental community intervention	4650 (62.96%)	Female: 48.1. Male: 51.4	Rural	CVD risk	Smoking; heavy alcohol consumption; salty diet; physical inactivity; overweight/obesity; high BP	In-person community meetings + mass-media/leaflets; clinic-based monthly visits for hypertensives	Health education; lifestyle counselling; pharmacotherapy for hypertensives; health-system strengthening in primary care
Oleson 2008 [51]	USA	Observational	787 (100%)	N/R	Rural and urban	Heart disease risk	Physical inactivity; unhealthy diet; overweight/obesity; smoking	In person (evening group sessions)	Health education/lifestyle counselling
Park 2024 [52]	USA	Observational	38 (100%)	N/R	Rural and suburban	CVD risk and stroke risk reduction	Physical inactivity; high sodium/poor diet; overweight/obesity; hypertension; hypercholesterolemia; prediabetes/diabetes; smoking	Hybrid: telephone and in person community programs	Counselling/coaching + education with subsidised access to community PA/nutrition programs
Perry 2007 [53]	USA	RCT	46 (100%)	I: 46 (10.9) C: 44 (11.2)	Rural	CVD risk	Physical inactivity; excess weight/obesity	Hybrid: In person and telephone	Behavioural counselling + group social support + structured walking
Pobee 1993 [54]	Ghana	Narrative review, epidemiological surveys, program evaluation	2645 (50.84%)	N/R	Rural and urban	Hypertension control for CVD risk and stroke risk reduction	Hypertension control, elevated BP; overweight/obesity (BMI); alcohol use	Hybrid: In person clinics & screenings; mass-media education (radio/TV) and print materials; professional meetings	Screening + pharmacologic management, patient/public education, clinician education, and health-system strengthening
Reiger 2015 [55]	Honduras	Evaluation	86 (80%)	59.6	Rural	Hypertension management for CVD risk and stroke risk reduction	Hypertension; medication non-adherence; overweight/obesity; low fruit/vegetable intake	In person (monthly group visits + CHW support)	Pharmacotherapy + group medical visits + CHW-led education/support; micro-insurance financing.
Rethorst 2024 [56]	USA	Community RCT	182 (100%)	57.2 (9)	Rural	CVD risk	Obesity/weight, hypertension (SBP), dysglycemia (HbA1c), hypertriglyceridemia, low physical activity, suboptimal diet	In person	Multilevel behavioral lifestyle program (education, exercise training, counselling/social support, community environmental change)
Safford 2024 [57]	USA	Cluster randomized clinical trial	1592 (62%)	58 (12)	Rural	Hypertension control for CVD risk reduction	Hypertension; medication non-adherence; diet; physical inactivity	Hybrid: telephone or in-person monthly + phone/email/video or materials & home BP monitors	Behavioral self-management coaching; practice quality-improvement facilitation; educational support & home BP monitoring
Sarrafzadegan 2006 [58]	Iran	Quasi-experimental	12,600 (49%)	N/R	Rural and urban	Primary prevention of NCDs, including CVD (IHD & stroke),	Tobacco, physical inactivity, unhealthy diet (e.g., replacing hydrogenated fats with oil; salt), stress; downstream risks (hypertension, hyperlipidemia, diabetes, obesity)	Hybrid public-health delivery: in-person community/clinical activities + mass media & policy/environmental changes	Health promotion/education, policy & environmental change, clinician education, and screening/follow-up for high-risk
Sarrafzadegan 2009 [59]	Iran	Community trial	Intervention: 6175 (51.3%) Control: 6339 (50.8%)	I: 38.6 (14.7), C: 39.1 (15.1)	Rural and urban	Primary prevention of NCDs, including CVD	Unhealthy diet, physical inactivity, smoking	Hybrid: in-person community/clinical activities + mass-media and policy/environmental changes	Health promotion/education, policy & environmental change, clinician education
Sarraf-Zadegan 2003 [60]	Iran	Community-based demonstration trial	12,600 (NR)	N/R	Rural and urban	Primary prevention of NCDs, including CVD (IHD & stroke),	Tobacco, unhealthy diet, physical inactivity, stress; downstream risks (BP, cholesterol, glucose, obesity)	Hybrid public-health delivery: in-person community/clinical activities + mass media, environmental and policy strategies	Health promotion/education; policy & environmental change; clinician education; screening & follow-up (high-risk)
Sherman 2007 [61]	USA	Pre–post intervention	75 (100%)	42.5	Rural	CVD risk	Physical inactivity, overweight/obesity, elevated BP, hyperchlesterolemia, hyperglycemia	Hybrid: in person, video, telephone	behavioural counselling/education with self-monitoring
Shoji 2022 [62]	Japan	Observational	1167 (60%)	55	Rural	Primary prevention of CHD and stroke	Blood pressure, lipids smoking, glucose/HbA1c & diabetes, BMI; medication use (antihypertensive, antidiabetic)	In person	Health education/brief counselling (awareness/behaviour change)
Sidebottom 2021 [63]	USA	Observational	5795 (“higher proportion female”)	53% 40–79 age band	Rural	CVD risk	Overweight/obesity, dyslipidemia (high total cholesterol), tobacco use, diabetes, physical inactivity	Hybrid: In person, telephone, broad community communications (mail, media, web, onsite)	Screening; behavioral counselling/education; health coaching; environmental/policy change
Siedner 2018 [64]	South Africa	Population-based cohort	1169 (70.5%)	N/R	Rural (with peri-urban/urban pockets)	CVD risk factor and stroke	Hypertension (primary); models considered BMI, diabetes, tuberculosis, geography as predictors	In-person	Screening plus referral/brief counselling
Ssetaala 2022 [65]	Uganda	Quasi-experimental prospective community study	486 (100%)	26.9 years	Rural	Maternal health services with screening of CVD risk factor (hypertension) and related conditions (anemia, HIV)	Hypertension; anemia; HIV	In-person	Counselling/education + point-of-care screening + referral, delivered by CHWs
Steenkamp 1991 [66]	South Africa	Quasi-experimental prospective clinical trial	7118 (53%)	Male 44.8 (12.8) Female 44.2 (12.4)	Rural	CVD risk	Smoking, hyperlipidemia, hypertension, inactivity, stress	In person	Health education
Sterling 2022 [67]	USA	Single-arm pre-post pilot	24 (92%)	43.6 (10.5)	Rural	CVD risk reduction	Obesity/overweight; dyslipidemia; systemic inflammation; low diet quality); physical inactivity	Hybrid remote: primarily online/virtual; in-person clinic assessments and drive-by food distribution	Lifestyle education/counselling, social support, structured diet/exercise; community-based and culturally tailored
Steyn 1993 [68]	South Africa	Quasi-experimental community study	2278 (53.9%)	Control male: 39.2 (15.8) Control female: 40.8 (14.8)	Rural	CVD risk to reduce CHD and stroke risk	Hypertension; also weight, alcohol, dietary salt/fat/cholesterol, exercise, smoking, and stress (non-drug management)	Hybrid: In-person, mass-media/community education	Screening & monitoring; lifestyle counselling/education; pharmacotherapy via primary care
Szeszulski 2024 [69]	USA	Process evaluation of a cluster randomized trial	182 (100%)	57.2 (9.0)	Rural	CVD risk	Obesity/overweight; physical inactivity; poor diet; BP, cholesterol (reported by participants/leaders)	Hybrid: In-person group classes at community sites; self-monitoring tech (Fitbit + scale) for immediate group	Behavioral lifestyle program (education, counselling, structured exercise) with social support and civic engagement; optional self-monitoring technology
Tessaro 2007 [70]	USA	RCT	262 (100%)	50.3	Rural	CVD risk	High dietary fat; low fruit/vegetable intake; nutrition-label literacy	In person (touch-screen computer in clinic waiting area)	Multimedia nutrition education (brief, computer-based)
Thorell 1994 [71]	Sweden	Longitudinal	215 (46.5%)	50–51	Semi-rural	Primary prevention of ischaemic heart disease via risk-factor	Smoking; blood pressure; serum cholesterol; overweight (BMI); lifestyle (diet, stress, exercise)	Hybrid In-person clinic screening and counselling; optional small-group video sessions	Screening + brief lifestyle counselling + referral (primary care)
Tian 2018 [72]	China	Qualitative descriptive study; cluster RCT	28 (70%)	41.0	Rural/remote	CVD risk management in primary care to prevent IHD/stroke	Hypertension/BP control; smoking cessation; dietary salt reduction; general CVD risk management	Hybrid: In-person primary care by village doctors, supported by a smartphone EDSS; home/clinic contacts; printed materials	Task-shifted primary-care management combining medication management, lifestyle counselling/education, and mHealth clinical decision support
van Assema 2006 [73]	Netherlands	Observational	1293 (82.1%)	47.5 (14.2)	Regional	CVD risk	High saturated fat intake; low fruit and vegetable intake; overweight/obesity (BMI distribution reported)	Hybrid: At home paper questionnaire with tailored mailed print feedback	Nutrition education, screening
Vedanthan 2019 [74]	Kenya	Cluster randomized trial	1460 (58%)	54.2 (16.4)	Rural	CVD risk to prevent IHD/stroke	Hypertension (counseling also addressed smoking, alcohol, physical activity)	Hybrid: In-person, mHealth support (smartphone arm)	Behavioral counselling/engagement via CHWs; mHealth decision support (smartphone arm)
Wang 2019 [75]	USA	Cost analysis and cost-effectiveness analyses; RCT	194 (100%)	N/R	Rural	CVD risk	Obesity/overweight; physical inactivity; inflammation; composite cardiovascular health	In-person group classes	Lifestyle education/counselling with structured exercise; multilevel community program
Weinehall 1999 [76]	Sweden	Quasi-experimental	2046 (NR)	N/R	Rural	CVD risk	Total cholesterol; SBP/DBP; smoking; BMI; lifestyle (diet, activity, alcohol, psychosocial)	Hybrid: In-person; community & media outreach; in-store food labelling	Screening & monitoring; brief lifestyle counselling/education; community health promotion
Wei 2021 [77]	China	RCT	28,130 (51%)	64.3 (6.1)	Rural	CVD risk particularly CHD and stroke	Hypertension and diabetes; smoking; salt and alcohol reduction; lipid management (statins) and antiplatelet therapy (low-dose aspirin)	Hybrid: In-person, telephone, text	Pharmaceutical therapy + lifestyle counselling + adherence support (primary care)
Williams 2004 [78]	USA	Pretest-posttest intervention	294 (100%)	N/R	Urban, rural	CVD risk	Elevated total cholesterol; elevated blood pressure; high BMI; high dietary fat intake; physical inactivity	Hybrid: In person + mailed letter follow-up	Screening plus brief lifestyle counselling/education
Wu 2024 [79]	China	Observational	8403 (52.77%)	63.5 (9.7)	Mixed (urban and rural, community-dwelling)	CVD risk	Hypertension and diabetes; lifestyle counselling on smoking, salt and alcohol reduction	In-person	Screening/monitoring plus health education/counselling (public health service package)
Xiao 2020 [80]	China	RCT	2912 (57.4%)	I: 57.7 (7.5), C: 58.1 (8.9)	Rural	CVD risk to prevent CHD/stroke	Hypertension; lifestyle risks: smoking, alcohol, high-fat/high-salt diet, physical inactivity; anthropometric/metabolic: BMI, waist, lipids, glucose, creatinine	Hybrid: In person (clinic follow-up and counselling) with ongoing in-person adherence monitoring; written materials provided	Pharmaceutical management + intensive lifestyle counselling/education with adherence monitoring
Zimmermann 2014 [81]	USA	Evaluation	162 (100%)	59.3 (11.8)	Rural	CVD risk	Diet quality; physical inactivity; CVD knowledge/self-efficacy (downstream: weight, cholesterol, BP)	In-person	Peer education/ health education (translation of evidence based messages)

N/R = not reported; SD = standard deviation; CVD = cardiovascular disease; IHD = ischaemic heart disease; NCD = non communicable disease; BP = blood pressure; SBP = systolic blood pressure; BMI = body mass index; HbA1c = glycated haemoglobin; HDL = high density lipoprotein cholesterol; LDL = low density lipoprotein cholesterol; PA = physical activity; CHW = community health worker

### Characteristics of included studies

The United States of America (34/62 studies) had the highest number of studies included, followed by China (4/62 studies), with the remaining studies from a combination of high-, middle- and low-income countries (Table 1). The sample size of the studies ranged from 24 participants to 12,600 participants. All studies targeted adults with 48 studies reporting on the specific age of participants. The mean age ranged from 26.9 years to 65.1 years, with the majority of studies having a mean age between 50 and 60 years.

Whilst all studies had to include females as a targeted population, 35% of studies were female-only (22/62 studies). The remaining studies included both females and males (40/62) (Table 1). Of the 40 studies that included females and males as targeted populations, 34 studies had a higher sample of women (> 50%) compared to men and 24 studies reported on gender-based differences in their results.

Most interventions were conducted in rural-only areas (43/62 studies; Table 1). Twelve interventions were delivered in both rural areas and semi-urban/urban/suburban areas; two interventions were delivered exclusively in regional settings; one was implemented in a remote-only setting, and the remaining interventions were based across a combination of rural, regional, remote, and semi-rural areas.

Of the 12 interventions that were delivered in both rural areas and semi-urban/urban/suburban areas, eight of these delivered interventions to both males and females.

Six studies reported utilising community and end-users input to design an intervention [25, 29, 35, 67, 76, 81]. Ten studies reported on professional input in the design of the intervention, such as nurses, pastors, community leaders, academic/research institutions, and stakeholders [29, 32, 36, 43, 48, 49, 54, 74, 76, 82].

### Cardiovascular disease sub-types and risk factors

All included studies focused on the primary prevention of CVD through addressing a combination of CVD risk factors. However, some studies were more specific in the sub-type of CVD that the intervention was trying to prevent, including stroke, coronary heart disease or ischaemic heart disease, and hypertension (Table 1).

Interventions targeted multiple CVD risk factors simultaneously. The most common risk factor targeted in interventions was hypertension (*n* = 41/62), followed by obesity (*n* = 39/62) and poor diet (*n* = 37/62). Other risk factors targeted included physical inactivity (35/62 studies), smoking (25/62 studies), high cholesterol (22/62 studies), alcohol intake (11/62 studies), and stress (8/62 studies) (Table 1).

For studies with female-only populations (22/62 studies), 14 studies addressed obesity, 13 studies addressed physical inactivity, 11 studies each addressed hypertension, and diet, and 5 studies addressed tobacco smoking. No female-only studies addressed stress.

### Intervention modality, type, and design

Thirty-four studies delivered their intervention in-person only, and mostly in a group setting (19/34 studies; Table 1). Twenty-eight studies delivered their intervention combining in-person delivery and alternate modalities. Modalities included online; telephone, email, print media, and text messaging.

Overall, most studies implemented interventions that combined multiple approaches to reduce CVD risk (*n* = 60/62 studies), with education being the most common approach included in interventions (*n* = 50; Table 1). The content focused on in these educational interventions included; diet, physical activity, health, lifestyle, and peer-to-peer education. Education was often combined with health coaching and/or counselling by clinicians (*n* = 25) and screening or monitoring (*n* = 13). Exercise was another type of intervention included in studies (*n* = 15) as well as pharmacotherapy, medication management, or chronic disease management (*n* = 10). Other intervention types included environmental changes (*n* = 6), social support (*n* = 5), policy change (*n* = 4), and mHealth (*n* = 3).

For studies that targeted females only (*n* = 22/62), education was also the most common approach (*n* = 18), followed by health coaching and/or counselling (*n* = 12) and exercise (*n* = 12; Table 1).

Three studies involved community input/participatory research in the design of their intervention [25, 29, 67]. Seven studies designed their intervention with professional or clinical input [29, 32, 43, 48, 49, 74, 82].

### Outcomes measured

#### Qualitative

There were five qualitative studies and four mixed-methods studies with a qualitative component. All studies included a combination of outcome measures. These outcome measures included participant satisfaction and perceived effectiveness or usefulness of the intervention (*n* = 4) [22, 46, 69, 72], participant knowledge (*n* = 5) [25, 45, 54, 69, 81] and barriers and facilitators to the intervention (*n* = 3) [46, 52, 72].

Three qualitative studies [45, 46, 52] and three mixed methods studies [25, 69] were targeted at female-only populations. One study [45] noted pre and post intervention knowledge gains in relation to: age as a risk factor for heart disease; significance in heart disease as a cause of death in females; and jaw, neck, or arm pain as a symptom of a heart attack. One [52] of these studies reported that demanding schedules and caregiving duties were a barrier to receiving intervention and another highlighted cost as a barrier to behaviour change [46]. Specifically, respondents noted the cost of formalised physical activity programs or gym memberships as a barrier to partake in exercise. Manuel, et al. [46] also reported that participants valued “hands on elements” in the intervention where grocery-store tours allowed females to translate their knowledge into real world experiences.

One qualitative study that was targeted at males and females [72] reported that the current financial incentive provided to village doctors would not “motivate them” to provide preventive care.

#### Qualitative: rurality

Two qualitative studies [46, 52] recruited participants from both rural and urban/suburban areas. Neither, however, reported on health outcomes following intervention delivery that were stratified by place of residence. Instead, authors highlighted contextual issues, noting that where access to healthcare services is limited in rural areas, alternative models of intervention delivery, such as telehealth, may be required for interventions to be successful [46]. It was also identified by participants that distance to food outlets acted as a barrier to receiving the intervention [52]. Both studies exclusively targeted women.

#### Quantitative

Quantitative studies (*n* = 53) measured a combination of outcomes. Most quantitative studies (*n* = 30) [21, 23, 29, 31–33, 35, 36, 38, 39, 41, 47–50, 56, 57, 60, 64, 65, 68, 71, 74, 77, 78, 80, 82, 83] measured blood pressure, followed closely by anthropometrics (e.g., weight, body mass index, body measurements) (*n* = 29) [20, 21, 29, 33–35, 37–39, 41–43, 47, 50, 51, 53, 56, 60, 63, 64, 67, 73, 75, 76, 78, 80, 84]. Of these studies, 19 [23, 24, 26, 31, 33, 34, 36, 47, 50, 56, 57, 68, 71, 74, 76, 77, 80, 82, 83] studies reported reductions in either systolic or diastolic blood pressure after receiving the intervention and 12 studies reported reduction in anthropometric measurements [20, 24, 27, 31, 33, 37, 39, 43, 47, 56, 67, 80].

Twenty studies measured self-reported dietary intake [44, 48, 50, 58, 59, 67, 70, 71, 73, 78] with 13 studies [20, 26, 28, 31, 37, 42, 44, 48, 59, 67, 69, 78, 84] noting improvements after the intervention was delivered. Eighteen studies measured self-reported physical activity levels [26, 28, 31, 35, 37, 39, 42–44, 47, 50, 51, 53, 58, 59, 61, 67, 84] and seven of these reported improvements in physical activity [28, 37, 42, 44, 48, 67, 84]. Only three (of 11) studies noted a reduction in smoking behaviours [58, 59, 66] and one (of 5) in alcohol consumption [50]. Twelve of the sixteen studies where lipids were an outcome saw an improvement in participant cholesterol readings [20, 24, 26, 33, 37, 39, 47, 67, 71, 76, 78, 80]. Other pathology measures assessed included urine and glucose) [23, 29, 33, 35, 39, 41, 56, 61, 80].

#### Quantitative: Gender differences

For the 40 studies that had male and female samples, 24 of these reported on gender -based differences in their results. The gender-differences reported were varied, and not all studies noted differences in their primary and secondary outcomes. For example, one study noted pre-intervention gender differences [24] and not post-intervention. Another study [82] reported on differences in drop-out rates (29% in males 61% in females).

Compared to males, females had greater improvement in medication adherence (females 45.6%, males 31.2%) [80]. Females also demonstrated greater CVD risk reduction [62, 76] and fruit and vegetable consumption [73]. Improvements were also greater for hypertensive control (mean reduction of 7.5mmHg in systolic blood pressure in women compared to 5.6mmHg) [68] and blood pressure reduction [50]. Females had a higher probability of linkage to care [64, 85] as well as higher response rates to surveys [23, 54] compared to males. Suggested reasoning for some of these differences include the implementation strategy targeting females [36], continuing trends in public health engagement in Sub-Saharan Africa [39] and cultural acceptability of males smoking compared to females in Vietnam [58, 59].

Compared to women, men were reported to have greater improvements in stroke incidence reduction [58]. The authors posit that these findings may reflect higher baseline risk in men. Men also had greater daily smoking rate reductions (*n* = 2) [58, 59] with a number of theories as to why suggested including: small and biased follow-up samples, intervention design focusing on multiple risk factors rather than smoking (low at baseline), national anti-smoking campaigns mainly targeting males, and rising secular smoking trends among Iranian women and youth [58]. Furthermore, men attended more group-based lifestyle intervention sessions [39], and had greater physical activity levels [58] post intervention.

#### Quantitative: rurality

Six quantitative studies [23, 51, 58, 64, 78, 79], and one mixed methods study [54] reported on outcomes related to differences in rurality. Four studies found rurality had an impact on intervention reach and CVD risk outcomes compared to interventions delivered in urban areas: Oleson, et al. [51] found distance from the intervention site played more of a role for those driving rurally compared to those living in urban areas; Siedner, et al. [64] found an association between distance from clinic and linkage to hypertension care, where linkage to care increased as distance to clinic increased; results from Wu, et al. [79] showed Health-Related Quality of Life improvements post intervention were less significant in rural dwellers compared to urban dwellers; and Williams, et al. [78] reported dietary and cholesterol risk were higher in rural areas post intervention, compared to urban areas.

One study [23] noted that smaller villages had greater reductions in urinary sodium compared to larger, “semi-urban” villages, after receiving the intervention. It further found that in rural villages, men and women had similar response rates, but in the semi-urban villages, men were less likely to respond compared to women. Pobee, et al. [54] also noted that response rates were higher in rural Ghana compared to urban areas (73% compared to 97%). The study noted that overall, older men had lower response rates compared to older women and younger men, however response rates were not further stratified by rurality. Siedner, et al. [64] reported that women were more likely than men to link to hypertension care, particularly in urban areas. They suggest this may be because men in urban settings had higher employment rates, leaving them with less time and flexibility to attend clinics and seek care.

Another study [58] found that smoking increased after delivery of intervention, but this was more prevalent in urban areas.

## Discussion

This paper aimed to examine community-based primary care interventions targeting the primary prevention of CVD by addressing modifiable risk factors among women living in rural, regional, and remote areas. Most studies implemented interventions that were not tailored to women or men; however, these studies typically reported higher participation rates among women with only half reported on gender-based differences in their results. More than half of the studies were conducted exclusively in rural areas. All included studies focused on the primary prevention of CVD by addressing multiple risk factors, most often through a combination of intervention types. Hypertension was the most targeted risk factor overall, while education emerged as the predominant intervention strategy. In contrast, among gender -specific interventions for women, obesity was the most frequently addressed risk factor, with education likewise being the most utilised intervention type. Blood pressure and anthropometric measurements were the most reported health outcomes, and in-person delivery was the most common modality (noting that digital-only interventions were excluded). Among studies that examined gender differences, reporting was often limited in detail and provided only tentative explanations for the observed disparities. Studies examining differences by rurality were similarly limited in number and scope.

Gender neutral interventions are typically seen in community-based and primary-care CVD prevention programs, which are often designed for a whole community approach and can still be effective without gender-specific tailoring [86]. Such gender-neutral designs can therefore be more scalable, particularly in resource-limited rural, regional, and remote settings [87]. However, gender-specific interventions can close gaps in access, relevance, and outcomes that gender-neutral programs often miss. Given the long-standing disparities women face in CVD diagnosis and treatment compared to men [88], gender-specific interventions are essential to addressing these inequities.

For women, tailored intervention designs can address gender-specific risk factors and life events (e.g., hypertensive disorders of pregnancy, gestational diabetes, early menopause, and polycystic ovarian syndrome) that elevate long-term CVD risk but are not often integrated into generic prevention or follow-up care [89, 90]. Women-only prevention pathways such as the WISEWOMAN program have demonstrated risk-factor improvements that are cost-effective for underserved groups such as rural, regional, and remote communities [91] which supports the case for targeted, gender-specific intervention delivery in resource limited settings. Although this review aims to focus on women, we briefly acknowledge the continued need for gender-specific interventions (including men) for equitable prevention; however, implications are discussed primarily for women.

The review demonstrated that in interventions that targeted both men and women, women often had participation representing over 50% of the study sample. This may reflect the differences in healthcare seeking behaviours between men and women where women have higher primary care contact. For example, in Australia, women have higher annual general-practice attendance than men (88% vs. 80%) [92]. Women typically exhibit higher survey response rates, which may contribute to greater engagement with community health initiatives and research, potentially contributing to their greater participation in these studies [93, 94]. This finding further indicates that women demonstrate a strong willingness to engage with interventions, and that existing programs have been effective in facilitating their participation.

The studies included in this review also provided limited gender-based analyses of gender-neutral interventions. Such underreporting of gender-specific differences aligns with broader patterns of gender bias consistently documented in clinical trials and across the wider evidence base [95–97]. A recent Australian study highlighted the significant underrepresentation of women particularly in cardiology and nephrology research [98].

In-person delivery, particularly in group-based formats, can be effective for women because it integrates behaviour change with peer support and a sense of shared identity. This can boost intervention engagement, adherence, and longer-term outcomes [99]. Women specific, in-person group programs in rural communities such as the StrongWomen-Healthy Hearts [100] program and Strong Hearts, Healthy Communities Program 2.0 [101] show meaningful improvements in weight, waist circumference, fitness, diet quality, and CVD risk, with some effects maintained beyond the program period. Some studies have shown that remote delivered care, mobile health, and hybrid interventions can achieve similar clinical outcomes for some cardiac care, CVD prevention, and education programs [70, 102, 103], however in-person group settings remain valuable where social connection is a key driver of intervention uptake [104]. Further research is required to evaluate other patient outcomes, efficacy, and cost effectiveness of remote-delivered care [103].

Across the six quantitative studies (and one mixed methods study) that reported outcomes by rurality, four suggested that a participant’s geographic location modified both intervention reach and cardiovascular outcomes. This is also supported by two qualitative papers that note the potential barriers that geography places on individuals seeking to address CVD risk. The findings in this review are consistent with broader evidence, which shows that small increases in travel distance or time can reduce utilisation of health services and are associated with worse outcomes, particularly for rural and remote populations [105, 106]. This reinforces that physical accessibility remains a critical consideration when designing CVD prevention programs in rural settings.

Most interventions in this review combined multiple approaches to CVD prevention (e.g., lifestyle education, goal setting, self-monitoring, and health coaching/counselling), which is consistent with prevention guidelines and evidence showing that multi-component programs produce broader and more sustained improvements in CVD risk factors than single-component approaches (such as education-only interventions) [107, 108]. Furthermore, education is effective when paired with behavioural support such as health coaching and counseling as it aids in adherence, building knowledge, and increasing self-efficacy in the targeted individuals.

### Gaps in literature

The review critically appraises the limited use of co-design approaches in intervention development. This contrasts sharply with strong evidence that participatory and community-led approaches improve relevance, acceptability, equity, and outcomes of interventions. Providing local context is of particular importance for acceptability and update among rural women by considering local norms, geographic and resource constraints [109]. From an implementation-science perspective, early stakeholder engagement is linked to better implementation outcomes such as acceptability, appropriateness, adoption, fidelity, and sustainability [110]. Recent reviews however have found that co-design is often poorly reported and terms such as ‘co-design,’ ‘co-production,’ ‘consumer engagement’ and ‘public and patient involvement’ often conflated, with actual involvement ranging from a one-off consultation to in-depth, ongoing engagement [111]. By foregrounding this gap, the review underscores the importance of embedding community engagement and lived experience into future prevention initiatives.

The intersection of gender and geography is only superficially addressed in the existing literature and should be more explicitly examined in future studies and reporting, given its importance for understanding differential patterns in access to and outcomes of care.

### Strengths and limitations

This review synthesises a breadth of studies and brings together evidence on both gender and rural differences in community-based primary CVD prevention interventions. By examining these factors, the review offers a more nuanced understanding of equity issues in prevention, an angle that is not often explored in depth in existing literature. A key strength lies in the deliberate focus on gender differences. While many mixed-gendered studies fail to disaggregate findings by gender, this review highlights the implications of such omissions, drawing attention to the potential gender bias embedded within primary prevention research, supporting what has previously been documented [8]. By explicitly noting where evidence is lacking, the review identifies clear directions for future investigation.

A key limitation of this study is that many included studies did not report gender-based differences in intervention outcomes, which limited direct comparisons of intervention effectiveness between genders. Among studies that did examine gender-based differences, reporting was often limited in depth. Evidence on differences by rurality was similarly constrained, with few studies examining outcomes across rural, regional, and metropolitan contexts. Furthermore, exclusion of interventions delivered exclusively in metropolitan or urban areas limits comparative insights and may reduce understanding of how intervention effectiveness differs by geography. Restriction to English-language studies also introduces language bias and may under-represent evidence from non–English-speaking rural and remote contexts, including Indigenous and low- and middle-income country settings.

### Policy and research implications

Future research should routinely include gender-disaggregated analyses to address inequities and clarify differential responses to interventions, thereby providing more evidence as to whether gender -specific approaches are more effective gender-neutral models. Future policies should prioritise co-designed CVD prevention, particularly in rural, regional, and remote communities. To reduce the disproportionate burden among women, gender -specific, co-designed strategies developed with rural, regional, and remote women are needed to ensure interventions reflect women-specific contexts and local needs in resource-constrained settings. To strengthen real-world impact and relevance, studies should embed co-production and co-design methods throughout all stages of the research process, from problem definition through to implementation, and rigorously evaluate implementation outcomes. Transparent and accurate reporting is essential. Moreover, addressing the persistent gender bias in health research, including the underrepresentation of women in cardiology and related fields, remains critical to ensuring that prevention strategies are equitable, inclusive, and generalisable. For practice, the findings support locally adapted, women-centered programs that integrate education with sustained behavioural support and social connection, delivered through feasible primary-care and community platforms.

## Conclusion

This review examined community-based interventions for the primary prevention of CVD among women living in rural, regional, and remote areas. Across 62 studies, evidence favoured multicomponent approaches to CVD prevention, typically combining education with coaching/counselling, goal-setting and self-monitoring, over single-component approaches, with in-person, group-based delivery showing promise for engagement and behaviour change among women. Yet most programs were not gender-specific, and co-design was seldom. Women had higher sample sizes in mixed- gender samples, consistent with greater primary-care utilisation and research participation. However, this does not replace the need for interventions that address women-specific risks and rural barriers. Gender based analysis was also limited, reflecting ongoing gender bias in research. Future work should co-design interventions with rural, regional, and remote based women.

## Supplementary Information

Below is the link to the electronic supplementary material.

Supplementary Material 1

## Data Availability

Results of articles can be found in Table 1. See supporting material file for sample search strategy.

## Abbreviations

CVD: Cardiovascular disease
