# Supplementary material for: Community based interventions for the primary prevention of cardiovascular disease in women living in rural, regional and remote areas: a scoping review
Source: BMC Health Serv Res. 2026 Feb 17;26:391. doi: 10.1186/s12913-026-14192-z (PMC13015016; doi:10.1186/s12913-026-14192-z)

**Additional File 1: Supplementary Material**

**Supplementary Table 1: Sample search strategy**

Database(s): Medline complete, 1945 to June 23 2025
Search Strategy: Embase Emtree synonyms for keywords; plus using author keywords from gold standard articles (Scopus); MeSH terms for concepts; title and abstract

Concept 1: synonyms for CVD found in Embase Emtree, other keywords added plus Mesh terms for the following: CVD, CHD, Stroke, arrythmia, heart failure, deep vein thrombosis, Congenital heart disease, Myocardial infarction

| **#** | **Searches** | **Results** |
| --- | --- | --- |
| Concept 1: CVD | “angiocardiopathy” OR “angiocardiovascular disease” OR “cardiovascular complication” OR “cardiovascular diseases” OR “cardiovascular disorder” OR “cardiovascular disturbance” OR “cardiovascular lesion” OR “cardiovascular syndrome” OR “cardiovascular vegetative disorder” OR “complication, cardiovascular” OR “disease, cardiovascular” OR “major adverse cardiovascular event” OR “cardiovascular disease” OR CVD OR “heart attack” OR stroke or “heart failure” OR arrythmia OR MH "Cardiovascular Diseases+" OR MH "Heart Diseases+" OR MH "Coronary Disease+" OR MH "Rheumatic Heart Disease" OR MH "Pulmonary Heart Disease" OR MH "Carcinoid Heart Disease" OR MH "Heart Valve Diseases+" OR MH "Aortic Valve Disease+" OR MH "Myocardial Ischemia+" OR MH "Cerebrovascular Disorders+" OR MH "Stroke+" OR MH "Ischemic Stroke+" OR MH "Hemorrhagic Stroke" OR MH "Embolic Stroke" OR MH "Thrombotic Stroke+" OR MH "Coronary Disease+" OR MH "Heart Diseases+" OR MH "Rheumatic Heart Disease" OR MH "Pulmonary Heart Disease" OR MH "Carcinoid Heart Disease" OR MH "Coronary Artery Disease" OR MH "Heart Valve Diseases+" OR MH "Myocardial Ischemia+" OR MH "Aortic Valve Disease+" OR MH "Heart Failure+" OR MH "Heart Failure, Diastolic" OR MH "Heart Failure, Systolic" OR MH "Peripheral Arterial Disease+" OR MH "Peripheral Vascular Diseases+" OR MH "Arrhythmias, Cardiac+" OR MH "Heart Failure+" OR MH "Venous Thrombosis+" OR MH "Upper Extremity Deep Vein Thrombosis" OR MH "Heart Defects, Congenital+" OR MH "Heart Diseases+" OR MH "Rheumatic Heart Disease" OR MH "Pulmonary Heart Disease" OR MH "Carcinoid Heart Disease" OR MH "Heart Valve Diseases+" OR MH "Coronary Disease+" OR MH "Myocardial Ischemia+" OR MH "Aortic Valve Disease" OR MH "Myocardial Infarction+" | 3,155,738 |
| For AMED | (angiocardiopathy) OR (angiocardiovascular disease) OR (cardiovascular complication) OR (cardiovascular diseases) OR (cardiovascular disorder) OR (cardiovascular disturbance) OR (cardiovascular lesion) OR (cardiovascular syndrome) OR (cardiovascular vegetative disorder) OR (complication, cardiovascular) OR (disease, cardiovascular) OR (major adverse cardiovascular event) OR (cardiovascular disease) OR CVD OR (heart attack) OR stroke OR (heart failure) OR arrhythmia OR (coronary artery disease) OR (myocardial infarction) OR (ischemic stroke) OR (hemorrhagic stroke) OR (embolic stroke) OR (thrombotic stroke) OR (heart defect) OR (congenital heart disease) OR (peripheral arterial disease) OR (peripheral vascular disease) OR (venous thrombosis) |  |
| Concept 2: Community based care/primary prevention | (community based intervention) OR (community intervention) OR (community-based program*) OR (community delivered) OR (community health program*) OR (community health care) OR (primary care intervention*) OR (local health intervention*) OR (population health intervention*) OR (community support program*) OR (community outreach) OR (community prevention program*) OR (community health initiative*) OR (primary prevention) OR (health promotion) OR (preventive health services) OR (integrated health care) OR (community health services) OR (community-based participatory research) OR (primary health care) | 654,190 |
| For AMED | "community based intervention" OR "community intervention" OR "community-based program*" OR "community delivered" OR "community health program*" OR "community health care" OR "primary care intervention*" OR "local health intervention*" OR "population health intervention*" OR "community support program*" OR "community outreach" OR "community prevention program*" OR "community health initiative*" OR “primary prevention” OR "Community Health Services" OR "Community-Based Participatory Research" OR "Primary Health Care+" OR "Health Promotion+" OR "Preventive Health Services" OR "Delivery of Health Care, Integrated” |  |
| Concept 3: Geography | rural OR remote OR regional | 631,892 |
| Concept 4: Women | women OR woman OR female OR females | 2,716,022 |
| 5 | 1 and 2 and 3 and 4 | 520 |
| 6 | 5 and Limited to English language | 479 |

**Supplementary Figure 1: PRISMA flow diagram**


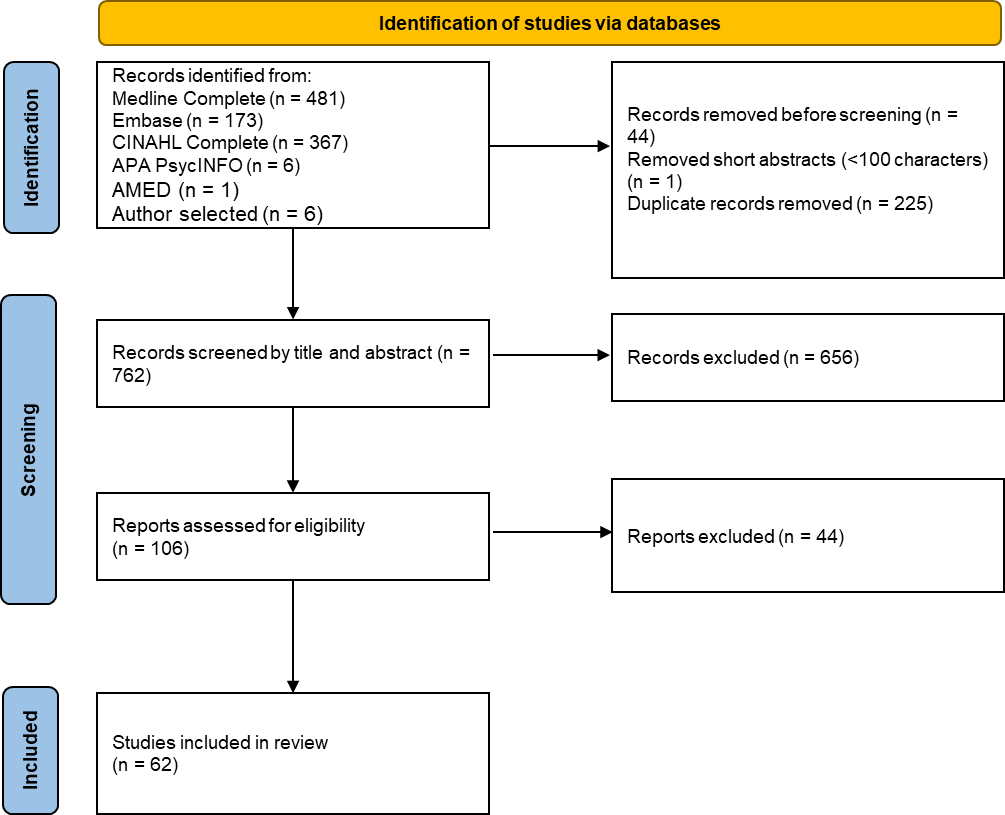

Supplement: Supplementary file 1 — Supplementary Material 1 [file 12913_2026_14192_MOESM1_ESM.docx]
